# Proteomic analysis of mare follicular fluid during late follicle development

**DOI:** 10.1186/1477-5956-9-54

**Published:** 2011-09-17

**Authors:** Somayyeh Fahiminiya, Valérie Labas, Stéphane Roche, Jean-Louis Dacheux, Nadine Gérard

**Affiliations:** 1 INRA, UMR 6175 Physiologie de la Reproduction et des Comportements, F- 37380 Nouzilly, France; 2 CNRS, UMR 6175 Physiologie de la Reproduction et des Comportements, F-37380 Nouzilly, France; 3 Université François Rabelais de Tours, UMR 6175 Physiologie de la Reproduction et des Comportements, F-37041 Tours, France; 4 Haras Nationaux, UMR 6175 Physiologie de la Reproduction et des Comportements, F-37380 Nouzilly, France; 5 INRA, UMR 6175 Plate-forme d'Analyse Intégrative des Biomarqueurs, Laboratoire de Spectrométrie de Masse, F- 37380 Nouzilly, France; 6 Institut de Génétique humaine du CNRS, UPR1142, 34396 Montpellier, France; 7 Plateforme de Protéomique Clinique, Hôpital Saint Eloi - Biochimie, 34295 Montpellier, France

**Keywords:** mare, ovary, follicular fluid, 2D-PAGE, hexapeptide ligand library

## Abstract

**Background:**

Follicular fluid accumulates into the antrum of follicle from the early stage of follicle development. Studies on its components may contribute to a better understanding of the mechanisms underlying follicular development and oocyte quality. With this objective, we performed a proteomic analysis of mare follicular fluid. First, we hypothesized that proteins in follicular fluid may differ from those in the serum, and also may change during follicle development. Second, we used four different approaches of Immunodepletion and one enrichment method, in order to overcome the masking effect of high-abundance proteins present in the follicular fluid, and to identify those present in lower abundance. Finally, we compared our results with previous studies performed in mono-ovulant (human) and poly-ovulant (porcine and canine) species in an attempt to identify common and/or species-specific proteins.

**Methods:**

Follicular fluid samples were collected from ovaries at three different stages of follicle development (early dominant, late dominant and preovulatory). Blood samples were also collected at each time. The proteomic analysis was carried out on crude, depleted and enriched follicular fluid by 2D-PAGE, 1D-PAGE and mass spectrometry.

**Results:**

Total of 459 protein spots were visualized by 2D-PAGE of crude mare follicular fluid, with no difference among the three physiological stages. Thirty proteins were observed as differentially expressed between serum and follicular fluid. Enrichment method was found to be the most powerful method for detection and identification of low-abundance proteins from follicular fluid. Actually, we were able to identify 18 proteins in the crude follicular fluid, and as many as 113 in the enriched follicular fluid. Inhibins and a few other proteins involved in reproduction could only be identified after enrichment of follicular fluid, demonstrating the power of the method used. The comparison of proteins found in mare follicular fluid with proteins previously identified in human, porcine and canine follicular fluids, led to the identification of 12 common proteins and of several species-specific proteins.

**Conclusions:**

This study provides the first description of mare follicular fluid proteome during the late follicle development stages. We identified several proteins from crude, depleted and enriched follicular fluid. Our results demonstrate that the enrichment method, combined with 2D-PAGE and mass spectrometry, can be successfully used to visualize and further identify the low-abundance proteins in the follicular fluid.

## Background

**Figure 1:**
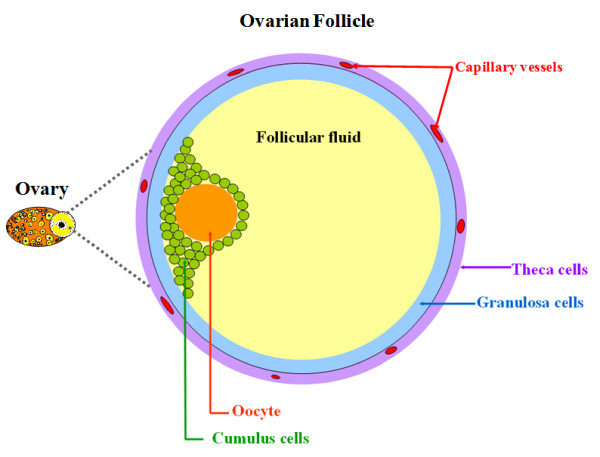
**Schematic picture of an ovarian antral follicle in mono-ovulant species**. cells are oocyte, cumulus, granulosa and theca. Follicular fluid accumulates into the center of the follicle and provides the micro-environment for growth, maturation and differentiation of follicular cells.

Follicular fluid accumulates into the follicle antrum starting with the early stage of follicle development. Plenty of evidence suggests that follicular fluid proteins originate from two sources: blood and surrounding somatic cell layers (granulosa and theca cells) (Figure 1). Earlier studies showed that the "blood-follicle barrier" is permeable for proteins below 500 kDa [1], and most proteins and other components easily pass through the basal lamina to enter the antrum, or escape towards circulating blood. Indeed, ovarian cells produce and secrete a number of soluble factors such as steroids, growth factors and other peptidergic factors into the follicular fluid [2]. The presence of all these factors is related to the metabolic activity of ovarian cells, and reflects the physiological status of the follicle. Furthermore, numerous studies have clearly demonstrated that these substances are essential for oocyte maturation and fertilization, granulosa cell proliferation and differentiation, and eventual ovulation and luteinisation [3]. However, the role in ovarian function played by many of these factors is still unknown. The study of follicular fluid components may contribute, to an understanding of the mechanisms involved in follicle differentiation and development.

Proteomic analysis of body fluids can have a helpful information for biomarkers discovery and treatment development [4]. To date, the most comprehensive proteomic analyses were performed on human follicular fluid. Spitzer et al. (1996) were first in studying comparatively the complex protein patterns of fluid from mature and immature human follicles [5]. Then, the study of Anahory et al. (2002) resulted in the identification of three proteins of human follicular fluid (thioredoxin peroxidase 1, transthyretin and retinol-binding protein) [6]. Three years later, Lee et al. (2005) identified four other proteins (hormone sensitive lipase, unnamed protein product 1, unnamed protein product 2, and apolipoprotein A-IV) in human follicular fluid [7]. Angelucci et al. (2006) performed an interesting and comprehensive proteomic study on human follicular fluid from women undergoing *in vitro *fertilization for male associated infertility [8]. The authors identified many proteins, mainly acute phase proteins and several proteins with antioxidant properties. Finally in 2010, Jarkovska et al. [9] used proteomic approach to study follicular fluid from women undergoing successful *in vitro *fertilization. They showed that proteins involved in the complement cascade may be associated with follicle/oocyte maturation. Proteomic analyses were also performed on follicular fluid of domestic animals, including bovine (5 proteins identified; Mortarino et al. 1999), porcine (53 proteins identified; Bijttebier et al. 2009) and canine (21 proteins identified; Fahiminiya et al. 2010) [10-12].

The ovarian physiology of the mare, compared to that of other mono-ovulant species, exhibits some uncommon features. Indeed, the ovulatory LH surge occurs as a progressive rise over several days, with a peak occurring one day after ovulation [13,14]. Moreover, the assisted reproduction techniques such as *in vitro *oocyte maturation, fertilization and embryo development have lower success rate than in bovine or human [15]. Investigations on the protein content of mare follicular fluid may provide useful information about the mechanisms underlying follicular development and oocyte maturation, and may lead to improvements in culture conditions and eventual success of assisted reproduction in this species.

We performed a proteomic analysis of the mare follicular fluid. Our study was divided into three parts: first, we hypothesized that proteins within mare follicular fluid differ from those in blood serum, and also could change during late follicle development leading to ovulation. For this purpose, we analyzed the global protein profile of both mare follicular fluid and serum, and then compared three different stages of the follicle development (early dominant, late dominant and preovulatory). Second, we used four different approaches of Immunodepletion and one of enrichment, to overcome the masking effect of high-abundance proteins present in the follicular fluid. Finally, we hypothesized that follicular fluid may contain a fixed set of proteins, regardless of species, and a variable set of proteins related to the specific physiological features of each species. Therefore, we compared our results with previous data obtained in mono-ovulant (human) and poly-ovulant (porcine and canine) species, to identify common and/or species-specific proteins.

## Results

### Proteomic analysis of crude mare follicular fluid

**Figure 2:**
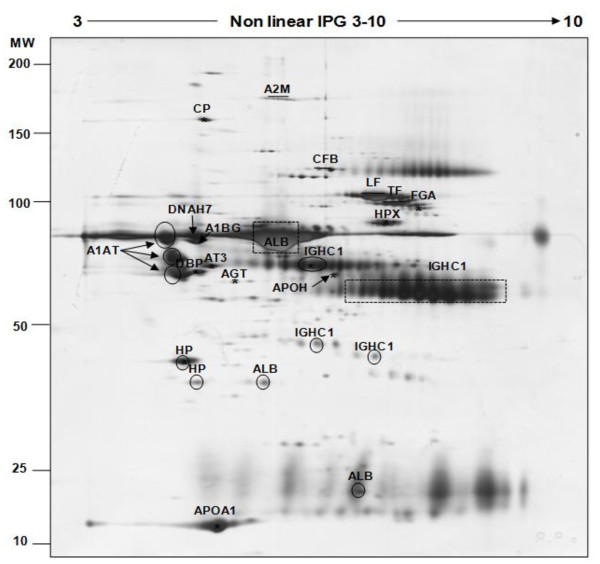
**Silver stained 2D-PAGE of crude mare follicular fluid**. 100 μg of proteins were applied to a non-linear IPG strip (pI 3-10) in the first dimension and separated on a SDS-PAGE (10%) gel in the second dimension (Molecular Weight: 10-200 kDa range). The positions of the high-abundance proteins albumin (ALB) and immunoglobulin heavy chain (IGHC1) are indicated by dark dotted rectangles or dark circles. All other identified proteins are named and their characteristics are shown in Table 1.

The 2D-PAGE method was used to characterize the protein profile of mare follicular fluid. The 2D patterns showed a total of 459 protein spots separated between pI (3-10) and molecular mass (10-200 kDa) intervals (Figure 2). Among them, 27 were excised from the gels and analysed by mass spectrometry. Assigned proteins are indicated on the 2D map (Figure 2) and are reported in Table 1. According to this result, 27 (100%) of the excised spots were identified, which represented 18 unique proteins. The high-abundant proteins, albumin (ALB) and immunoglobulin heavy chains (IGHC1) appeared as intense and large spots in protein patterns of mare follicular fluid (indicated by dotted rectangles in Figure 2). In addition, 4 (14.81%) proteins (albumin, immunoglobulin heavy chains, alpha-1-antitrypsin and haptoglobin precursor) were identified as multiple spots (indicated by dark circles in Figure 2).

**Table 1:** Identified proteins of crude mare follicular fluid separated by 2D-PAGE (pI: 3-10; MW: 10-200; silver staining)

Gene	**Accession N°**^**1**^	Protein name	**Score**^**2**^	Theoretical		**Peptide N°**^**3**^	**Sequence coverage (%)**^**4**^
						
				**pI**^**3**^	**Mr (Da)**^**3**^		
A1BG	gi|194216172	Alpha-1-B glycoprotein	374	8.74	68222	10	21
A2M	gi|194211675	Alpha-2-macroglobulin	1389	6.24	163911	21	25
AGT	gi|194206059	Angiotensinogen	50	7.06	70056	1	3
ALB	gi|76363596	Albumin	294	5.89	68494	31	43
ALB	gi|76363596	Albumin	112	5.89	68494	2	4
ALB	gi|399672	Preproalbumine	91	5.95	68554	2	4
APOA1	gi|3915607	Apolipoprotein A-I	105	5.2	30178	2	7
APOH	gi|149723623	Apolipoprotein H	33	8.43	38511	1	2
AT3	gi|179161	Antithrombin III	92	6.32	52585	2	5
DNAH7	gi|149699076	Similar to dynein, axonemal, heavy polypeptide 7 isoform 1	232	5.5	61446	5	11
CFB	gi|149732066	Complement factor B	141	6.75	85930	3	4
CP	gi|180249	Ceruloplasmin	48	5.29	97637	1	1
DBP	gi|73975213	Vitamin D-binding protein	39	5.2	52940	1	1
FGA	gi|3789960	Fibrinogen alpha	57	6.45	49477	2	4
HP	gi|149699777	Haptoglobin precursor	103	6.24	45176	5	9
HP	gi|149699777	Haptoglobin precursor	112	5.59	38441	3	9
HPX	gi|386789	Hemopexin	32	6.57	51512	1	1
IgG	gi|9858135	Immunoglobulin G1b	70	5.7	46904	2	10
IGHC1	gi|15020816	Immunogobulin gamma 1	30	7.68	37415	1	2
IGHC1	gi|15020816	Immunogobulin gamma 1	30	7.68	37415	1	2
IGHC1	gi|15020816	Immunogobulin gamma 1	30	7.68	37415	1	2
IGHC1	gi|15020816	Immunogobulin gamma 1	30	7.68	37415	1	2
LF	gi|13431954	Lactoferrine	54	8.66	77162	1	3
A1AT	gi|194225326	Alpha-1-antitrypsin	45	5.23	46913	2	3
A1AT	gi|194225326	Alpha-1-antitrypsin	398	5.31	46896	5	13
A1AT	gi|194225326	Alpha-1-antitrypsin	83	5.23	46913	3	5
TF	gi|3892525	Transferrin	219	8.6	6880	4	42

^1 ^**Accession N° **is an accession number from the NCBInr databases. ^2 ^Score is -10*Log (P), where P is the probability that the observed match is a random event, it is based on NCBInr database using the MASCOT searching program. ^3 ^**PI**: isoelectric point; **Mr**: Molecular weight; **Peptid N**°: Peptid number. ^4 ^Sequence coverage (%) is based on number of peptide masses matched in NCBInr databases.

#### Comparison of mare follicular fluid protein patterns with serum and between three stages of follicular development

**Figure 3:**
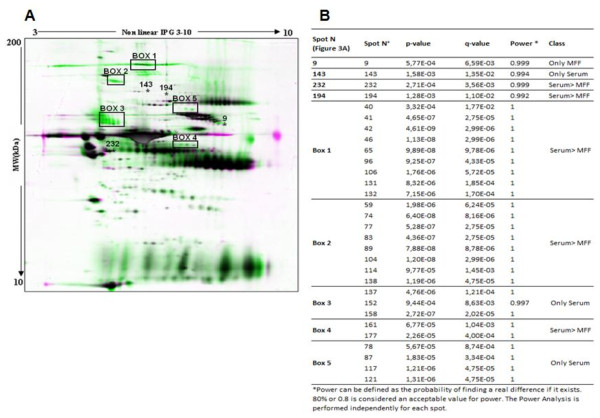
**Differentially expressed proteins between mare follicular fluid and serum**. (**A**) 2D-PAGE profile of mare follicular fluids (MFF) and serum after being matched by Progenesis Software. The reference gel is in green and all other 2D-PAGE from the same analysis is in Magenta. Thirty protein spots were identified as differentially expressed between follicular fluid and serum. (**B**) The complete list of 30 differentially expressed protein spots with related statistical value (p-value, q-value and power), provided by Progenesis software.

The 2D-PAGE method was applied to compare the protein patterns of mare follicular fluid samples collected from early dominant (size of follicles: 25.67 ± 0.67 mm, n = 3), late dominant (size of follicles: 34.67 ± 1.20 mm, n = 3) and preovulatory (size of follicles: 39.33 ± 1.76 mm, n = 3) follicles. The 2D patterns of protein expression appeared largely similar in the three stages, and the image analysis revealed no differentially expressed proteins during follicle development. Moreover, the patterns of protein expression between follicular fluid and serum appeared largely similar but some clear differences were obvious (Figure 3A). We observed 30 differential protein spots between mare follicular fluid and serum, one (spot N° 9) was present only in follicular fluid, eight spots were only in serum (boxes 3, 5 and spot n° 143) and 21 spots (boxes 1, 2, 4 and spots N° 232 and 194) were less intense in follicular fluid than in serum (Figure 3B). These spots were excised from the gels and analysed by mass spectrometry. The result of protein identification showed that the spots number 232 and boxes 1 and 2 correspond to antithrombin-III, alpha-2-macroglobulin and ceruloplasmin, respectively. Other spots were not identified positively in this study.

### Pre-fractionation of mare follicular fluid before proteomic analysis

#### Depletion of mare follicular fluid using four different Immunodepletion columns

**Figure 4:**
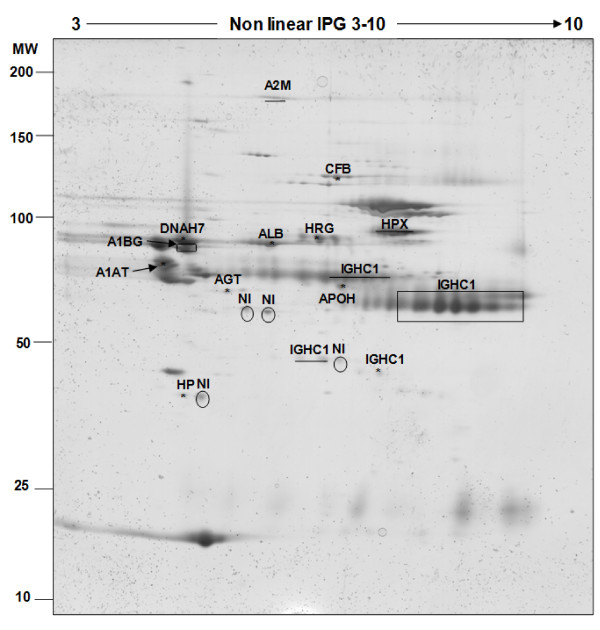
**Identification of proteins from depleted mare follicular fluid by MARS-6**. 100 μg of proteins were applied to a non-linear IPG strip (pI 3-10) in the first dimension and separated on SDS-PAGE (10%) (Molecular Weight: 10-200 kDa range). All identified proteins are named and their characteristics are shown in Table 2. Rectangle shows the common location of IGHC1 on the 2D-PAGE. NI: Not-Identified proteins.

In order to clear the follicular fluid from high-abundance proteins, and to attempt to visualize and identify some low-abundance ones, four Immunodepletion columns, developed to capture one (ProteomeLab IgY-HSA), six (MARS-6), twelve (ProteomeLab IgY-12) or twenty (ProteoPrep 20) high-abundance proteins from human serum (Additional file 1), were used in this study. Under our experimental conditions and considering protein assays, higher depletion efficiencies were obtained with the two IgY columns (81% for ProteomeLab IgY-12 and 75.5% for ProteomeLab IgY-HSA), than with the two other columns based on IgG (70.58% and 55.94% for MARS-6 and ProteoPrep 20, respectively). As shown in Additional file 2, the 2D-PAGE patterns of depleted fractions revealed that ProteomeLab IgY-HSA and MARS-6 were more efficient in eliminating albumin from mare follicular fluid than the two other columns tested. The lowest percentage of depletion efficiency observed for the ProteoPrep 20 column was confirmed by 2D-PAGE, since this column depleted neither ALB nor IGHC1 from the mare follicular fluid. Eighteen protein spots, preferentially chosen by localization, were excised from MARS-6 2D-PAGE (Figure 4) and analysed by mass spectrometry. Out of them, 77% (14/18) were successfully identified as 12 unique proteins (Table 2).

**Table 2:** Identified proteins of mare follicular fluid after depletion with MARS-6 and separation by 2D-PAGE (pI: 3-10; MW: 10-200; silver staining)

Gene	**Accession N° **^**1**^	Protein name	**Score**^**2**^	Theoretical		**Peptide N°**^**3**^	**Sequence coverage (%)**^**4**^
						
				**pI**^**3**^	**Mr (Da)**^**3**^		
A1AT	gi|194225326	Alpha-1-antitrypsin	398	5.31	46896	6	13
A1BG	gi|194216172	Alpha-1B-glycoprotein	374	8.74	68222	8	21
A2M	gi|194211675	Alpha-2-macroglobulin	1406	6.24	163911	25	26
AGT	gi|194206059	Angiotensinogen	50	7.06	70056	1	3
ALB	gi|76363596	Serum albumin precursor	112	5.89	68494	2	4
APOH	gi|149723623	Apolipoprotein H	33	8.43	38511	1	2
CFB	gi|149732066	B-factor, properdin	139	6.75	85930	2	4
DNAH7	gi|149699076	Dynein, axonemal, heavy polypeptide 7 isoform 1	232	5.5	61446	5	11
HP	gi|149699777	Haptoglobin precursor	112	5.59	38441	3	9
HPX	gi|149719403	Hemopexin	122	7.58	51324	3	6
HRG	gi|194222677	Histidine-rich glycoprotein	245	7.64	42955	5	16
IGHC1	gi|15020816	Immunogobulin gamma 1 heavy chain constant region	31	7.68	37415	1	2

^1 ^**Accession N° **is an accession number from the NCBInr databases.^ 2 ^Score is -10*Log (P), where P is the probability that the observed match is a random event, it is based on NCBInr database using the MASCOT searching program. ^3 ^**PI**: isoelectric point; **Mr**: Molecular weight; **Peptid N**°: Peptid number. ^4 ^Sequence coverage (%) is based on number of peptide masses matched in NCBInr databases.

Among these proteins, one (immunoglobulin gamma 1 heavy chain constant region) was identified in 3 different locations on the gel (rectangle in Figure 4 shows the common location of IGHC1 on 2D-PAGE). In addition, one single protein (histidine-rich glycoprotein) was identified from an excised spot in the region initially occupied by albumin, which clearly shows the masking effect of albumin as a high-abundant protein.

#### Enrichment of mare follicular fluid using Hexapeptide ligand library

**Figure 5:**
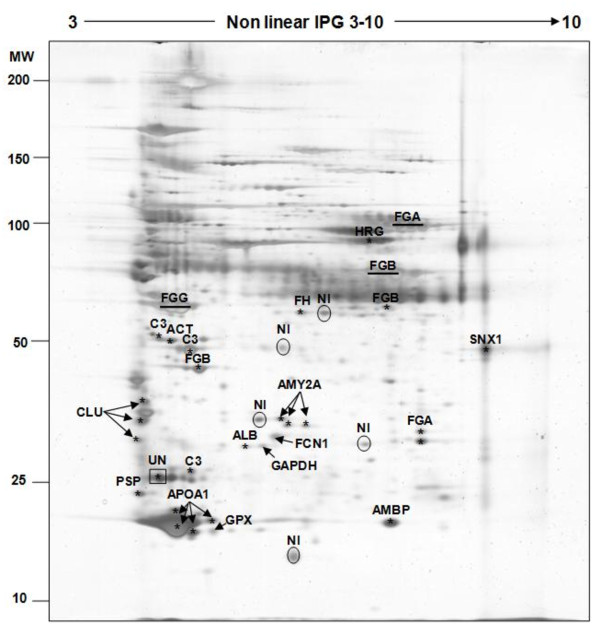
**Identification of proteins from enriched mare follicular fluid by hexapeptide ligand library column**. 100 μg of proteins were applied to a non-linear IPG strip (pI 3-10) in the first dimension and separated on SDS-PAGE (10%) (Molecular Weight: 10-200 kDa range). All identified proteins are named on the figure and their characteristics are shown in Table 3. NI: Not-Identified proteins.

Hexapeptide ligand library (marketed as ProteoMiner^®^) column was used to enrich mare follicular fluid and 2D-PAGE was performed to visualize the proteins between 3-10 pH intervals (Figure 5). Thirty-seven protein spots were excised from the gels and analysed by mass spectrometry. Thirty-three (89%) were successfully identified, which correspond to 17 unique proteins (Table 3) and one unnamed protein product (UN: accession number = gi|28317; indicated by dark rectangle in Figure 5). In addition, 5 protein spots (indicated by dark circles in Figure 5) were not identified, most probably due to the low intensity of proteins resulting in low sequence coverage insufficient for identification.

**Table 3:** Identified proteins of mare follicular fluid after enrichment by hexapeptide ligand library and separation by 2D-PAGE (pI: 3-10; MW: 10-200; silver staining)

Gene	Accession **N° **^**1**^	Protein name	**Score**^**2**^	Theoretical		**Peptide N°**^**3**^	**Sequence coverage (%) **^**4**^
						
				**pI **^**3**^	**Mr (Da)**^**3**^		
ACT	gi|6626	Actin	55	5.29	41779	1	2
ALB	gi|126723507	Preproalbumin	188	5.95	68554	4	7
ALB	gi|2492797	Albumin precursor	28	5.85	67837	1	1
AMBP	gi|149738520	AMBP protein precursor	101	6.82	38985	2	5
AMY2A	gi|178585	Alpha-amylase	78	6.32	57714	1	3
AMY2A	gi|178585	Alpha-amylase	37	6.32	57714	1	3
AMY2A	gi|178585	Alpha-amylase	106	6.32	57714	1	3
APOA1	gi|149716548	Apolipoprotein A-I precursor	883	5.66	30312	13	50
APOA1	gi|149716548	Apolipoprotein A-I precursor	675	5.66	30312	13	44
APOA1	gi|149716548	Apolipoprotein A-I precursor	900	5.66	30312	17	62
APOA1	gi|149716548	Apolipoprotein A-I precursor	281	5.66	30312	6	25
C3	gi|73987236	Complement C3 precursor	85	6.95	173988	1	1
C3	gi|116597	Complement C3 precursor	53	6.12	186342	1	0
C3	gi|554423	Complement component C3	179	5.73	31844	3	17
C3	gi|554423	Complement component C3	86	5.73	31844	2	8
CLU	gi|126352584	Clusterin	43	5.77	52121	1	2
CLU	gi|126352584	Clusterin	248	5.77	52121	3	12
CLU	gi|126352584	Clusterin	240	5.77	52121	4	12
FCN1	gi|194226003	Ficolin 1	156	5.37	34486	4	14
FGA	gi|3789960	Fibrinogen A-alpha chain	65	6.45	49477	1	2
FGA	gi|3789960	Fibrinogen A-alpha chain	65	6.45	49477	2	4
FGA	gi|3789960	Fibrinogen A-alpha chain	695	6.45	49477	10	37
FGB	gi|149698358	Fibrinogen beta chain precursor	502	8.53	55398	6	26
FGB	gi|149698358	Fibrinogen beta chain precursor	409	8.53	55398	8	28
FGB	gi|149698358	Fibrinogen beta chain precursor	137	8.53	55398	2	6
FGB	gi|149698358	Fibrinogen beta chain precursor	125	8.53	55398	2	6
FGG	gi|1916266	Fibrinogen-gamma	69	4.78	8740	2	62
FH	gi|194227377	Complement regulator factor H	95	7.52	140649	2	1
GAPDH	gi|31645	Glyceraldehyde-3-phosphate dehydrogenase	38	8.26	36031	1	4
GPX	gi|7262515	glutathione peroxidase	144	5.78	8893	2	37
HRG	gi|194222677	Histidine-rich glycoprotein	227	7.64	43003	4	12
PSP	gi|126352355	Parotid secretory protein	79	4.75	26882	2	9
SNX1	gi|149711176	Nexin-1 isoform 1	75	9.53	44065	1	3
UN	gi|28317	Unnamed protein product	84	5.17	59492	1	2

^1 ^**Accession N° **is an accession number from the NCBInr databases.^2 ^Score is -10*Log (P), where P is the probability that the observed match is a random event, it is based on NCBInr database using the MASCOT searching program. ^3 ^**PI**: isoelectric point; **Mr**: Molecular weight; **Peptid N**°: Peptid number^4 ^Sequence coverage (%) is based on number of peptide masses matched in NCBInr databases.

In addition to the proteins identified in crude mare follicular fluid, this strategy allowed us to identify other proteins which were either cytosolic (ACT, SNX1, GAPDH, GPX) or secretory (AMBP, FCN1, CLU, C3, FGG, FGB, FH and PSP). Furthermore, 3 proteins (Fibrinogen β, complement regulator factor H and AMBP) were identified in the region initially occupied by heavy and light immunoglobulin chains.

**Figure 6:**
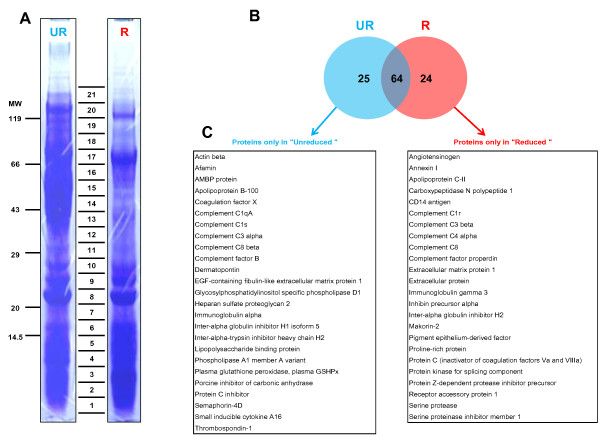
**1D-PAGE separation of mare follicular fluid proteins after enrichment by hexapeptide ligand library column under unreduced (UR) and reduced (R) conditions**. (**A**) 21 bands were cut in each lane from 1D-PAGE (CBB staining) and the proteins were identified by mass spectrometry. The result of identification is summarized in Additional file 3 for reduced conditions and Additional file 4 for unreduced conditions. (**B**) Venn diagram highlights the non-overlapping and common proteins identified in both conditions. (**C**) Tables show the list of non-overlapping proteins in each condition (alphabetical order).

The result of the shotgun separation method (1D-PAGE-LC) that we performed on the enriched fraction of mare follicular fluid is shown in Figure 6A. The electrophoretic separation patterns of both reduced and unreduced conditions were similar, with estimated molecular weights ranging from less than 14 kDa to greater than 100 kDa. Both lanes were cut into 21 fragments, subjected to trypsin digestion and analyzed by mass spectrometry.

The major band visualized in unreduced conditions with molecular weights from 40 to 60 kDa (excised bands from 13 to 16) was not present in reduced conditions. The result of protein identification in this region showed that the dominant proteins were mainly immunoglobulin gamma 1 heavy chain (37 kDa), histidine-rich glycoprotein (42 kDa), apolipoprotein A-IV (43 kDa), alpha-1-antitrypsin (46 kDa), fibrinogen A-alpha (49 kDa), fibrinogen gamma (51 kDa), vitronectin (54 kDa), fibrinogen beta (55 kDa), EGF-containing fibulin-like extracellular matrix protein 1 isoform (55 kDA) and albumin/preproalbumin (68 kDa). In addition, we identified the same number of proteins (n = 88) from the 21 excised bands in both conditions (Additional files 3 &4). Sixty-four were identified under both conditions, 24 proteins were identified only under reduced conditions, and 25 only under unreduced conditions (Figure 6B). Some of these 49 uncommon proteins were different isoform of the same family proteins (complement factors, immunoglobulin or inter-alpha globulin). Altogether, we were able to separate and identify proteins with a molecular mass of only 3 kDa using unreduced conditions (plasma glutathione peroxidase), and 11 kDa under reduced conditions (Apolipoprotein C-II).

### Subcellular and functional annotation of mare follicular fluid proteome

**Figure 7:**
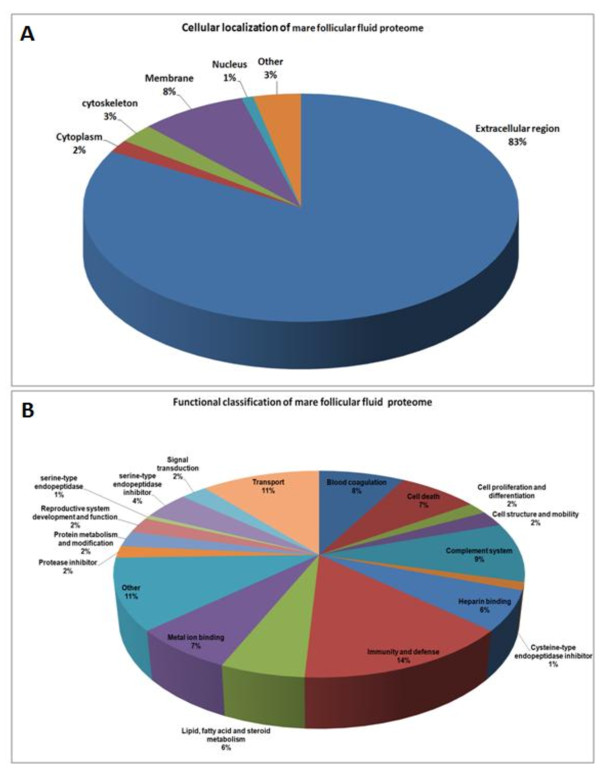
**Annotation of mare follicular fluid proteome**. (**A**) Subcellular localization and (**B**) functional classification of all proteins identified in mare follicular fluid.

Bioinformatic analysis was performed on the mare follicular fluid proteome, i.e. on all proteins that we identified in the present study, in order to determine their subcellular localization and their molecular function. The analysis of subcellular localization demonstrated that as many as 83% of proteins were localized in the extracellular region (Figure 7A). The rest were localized in the intracellular compartment, including cytoplasm, cytoskeleton, plasma membrane or nucleus.

The functional annotation showed that proteins were involved in various molecular functions (Figure 7B). A total of 18 groups of molecular functions were classified. The two most abundant include 25% of the proteins, involved either in immunity and defense (14%) or transport (11%). The complement system and blood coagulation group together represent 17% of the proteins. In contrast, proteins involved in lipid-fatty acid-steroid metabolism or in reproductive system development and function only represent 6% and 2%, of the identified proteins, respectively. In addition, a substantial number of identifications concerned proteins with inhibitory function of serine proteases (e.g. plasma serine protease inhibitor and alpha-1 antitrypsin), cystein proteases (fetuin-B) and serine-type endopeptidase (inter-alpha-trypsin inhibitor heavy chain H1/H2/H4, protein AMBP, protein Z-dependent protease inhibitor). The list of proteins with their function and subcellular localization were summarized in Additional file 5.

### Overlapping of mare follicular fluid proteins with those from three other species

**Figure 8:**
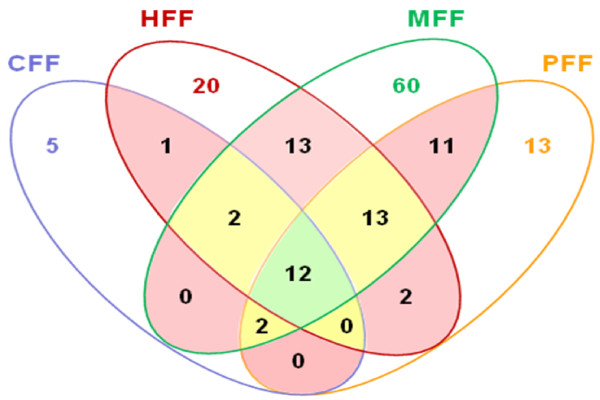
**Overlap of identified proteins in follicular fluid of four different species**. Green (12 proteins) and yellow (17 proteins) areas contains the proteins which are present in all or 3 out of 4 species (CFF: **C**anine, HFF: **H**uman, MFF: **M**are and PFF: **P**orcine **F**ollicular **F**luid), respectively.

We compared our results with other follicular fluid proteomic studies performed in three mammalian species including canine, human and porcine (Table 4) [8,9,11,12]. The Venn diagram (Figure 8) revealed that a set of 12 proteins were common to the four species. In addition, 17 proteins were present in at least three out of four species. We also observed that 60, 20, 13 and 5 proteins were identified only in mare (MFF), human (HFF), porcine (PFF) and canine (CFF) follicular fluid, respectively. The list of these species specific proteins (Table 5) showed that some of them were various fragment of high abundant proteins like immunoglobulins, complement factors or apolipoprotein. This may be due to various experimental procedures on the samples. Interestingly, the proteins related to reproduction (inhibins), were identified only after enrichment of mare follicular fluid (present study). Finally, all the identified proteins from follicular fluid of the four species were combined and the protein list was improved from 113 (our study) to 154 (Additional file 6).

**Table 4:** Overview of studies performed on follicular fluid of four different species

Study	Follicular fluid samples	Part of sample analyzed	Separation method	MS method
This study	Mare (MFF)	crude, depleted and enriched mare follicular fluid from normal follicles	2D-PAGE 1D-PAGE	nano-LC-MS/MS
Fahiminiya et al. (2010)	Canine (CFF)	crude canine follicular fluid from normal follicles	2D-PAGE	nano-LC-MS/MS
Angelucci et al. (2006)	Human (HFF)	crude follicular fluid from normo-ovulatory women undergoing assisted reproduction techniques due to a male infertility factor	2D-PAGE	MALDI-TOF-MS
Jarkovska et al. (2010)	Human (HFF)	depleted follicular fluid of women undergoing successful IVF	2D-PAGE ProteomeLab PF 2D	MALDI-TOF-MS
Bijttebier et al. (2009)	Porcine (PFF)	crude porcine follicular fluid of normal follicles	iTRAQ labeling	LC ESI-Q-TOF MS/MS

For each study the following information is presented: 1) follicular fluid derived from specific species 2) the nature of the samples, used in the study (physiological status and whether the study focused on depleted, enriched or crude follicular fluid) 3) the methods used to separate proteins and peptides and 4) MS method used for analysis.

**Table 5:** The list of species specific proteins presented in Figure 8

Follicular fluid	Proteins
Canine (CFF)	ACT, HPR, HV01, HV02, AZGP1
Human (HFF)	ALPPL2, ANXA4, CAT, EIF4E, SOD3, FCN3, GSTA2, GSTP1, GCHFR, HSPB1, IGFBP1, LMNA, LRG1, LDHB, NME1, PDIA3, APCS, TPI1, UCHL3, ZNF229
Porcine (PFF)	ORM1, AFP, ANGPTL2, APOC3, Cd5l, F9, COL7A1, CST7, gpr107, HBB, SERPIND1, ApoN, TGFBR2
Mare (MFF)	ACTB, A1BG, ANXA1, APOB, APOC2, C4BPA, CESdD1, CPN1, CCL16, F10, F12, F13A1, C1QA, C1R, C5, C8A, C8G, CFP, CRISPLD2, DPT, DNAH7, EFEMP1, ECM1, FETUB, FBLN1, FCN1, GPX3, GAPDH, HLA-B, IGHG3, IgG, IGHC1, IgG6, IgG7, **INHA, INHBB**, KNG1, LTF, LRRC15, LBP, LYZL1, MFAP4, CD14, AMY2A, Psp, LCAT, GPLD1, PLA1A, SERPINF1, SERPING1, SERPINA5, MKRN2, Cdk12, SERPINA10, REEP1, SEMA4D, HTRA1, SNX1, THBS1, PROC

## Discussion

Follicular fluid is the essential environment for growth and maturation of both ovarian somatic and germ cells. It contains substances implicated in cell differentiation, gamete quality and rupture of the follicular wall. One can reasonably expect that the determination of its protein composition will contribute to a better understanding of ovarian physiology and possibly to the regulation of follicular growth and development. We performed a proteomic analysis of the mare follicular fluid. Our study was split into three parts: first, we hypothesized that proteins of mare follicular fluid differ from serum and change during the late follicle development leading to ovulation. For this purpose, we analyzed the global protein profile of mare follicular fluid and compared the profiles of follicular fluid samples collected at three stages of final maturation of the follicle, as well as the serum profile. Second, we used different approaches of Immunodepletion and enrichment to deprive the follicular fluid of high-abundant proteins, to reduce their masking effect. Finally, we hypothesized that a fixed set of proteins would be present in follicular fluid regardless of the species, as well as a variable set of proteins which depends on the reproductive specificity of each species. Therefore, we compared our results with previous data obtained in mono-ovulant (human) and poly-ovulant (porcine and canine) species, in order to identify common and/or species-specific proteins.

### Crude mare follicular fluid proteomic analysis

To reach our first objective, we used 2D-PAGE to characterize the global protein profile of mare follicular fluid, and mass spectrometry to identify some of the proteins. By combining these two techniques, we were able to identify 27 protein spots in the mare follicular fluid, corresponding to 18 unique proteins. The majority of identify proteins belonging to the high-abundance serum proteins (fragments of albumin, immunoglobulin G, transferrin, fibrinogen, antitrypsin, haptoglobin, apolipoprotein and ceruloplasmin). All of them had been identified previously in the follicular fluid from bovine [10], canine [11], human [8] and porcine [16]. Furthermore, four proteins, including albumin, immunoglobulin heavy chains, alpha-1-antitrypsin and haptoglobin precursor, were identified in multiple spots, probably due to post-translational modifications and processing.

Many evidence supports the role of pituitary gonadotropins (FSH and LH) in regulating numerous proteins implicated either in selection, ovulation or luteinization of the ovarian follicles [3]. Although most of them display a cellular localization, some have been localized in follicular fluid such as extracellular matrix glycoproteins [17,18], proteinases [19,20] and their inhibitors [21,22]. In the present investigation, we attempted to visualize and identify proteins in mare follicular fluid that may be modulated during follicle selection and in response to the increase in circulating LH level. The comparison of computerized protein patterns of mare follicular fluids collected at different physiological stages revealed no differentially expressed proteins. In fact, the 2D proteins patterns were almost similar among groups, and the high-abundance proteins, were observed as dominant intense spots on the 2D-PAGE. This result was in accordance with an earlier study in which no difference was observed in protein patterns of human follicular fluid derived from mature and immature follicles [5]. One hypothesis could be that during follicle growth and development, the follicular cell layers become more vascularized and permeable to serum proteins. As a consequence, serum proteins may pass thought the basal lamina and enter into the follicle antrum. These proteins are present at high concentration in the follicular fluid, and can mask the less abundant proteins that are locally produced by follicle cells.

In order to identify some proteins produced locally by follicular cells, we also compared the 2D-PAGE patterns of crude follicular fluid with matching serum samples. Thirty differential proteins spots were observed, from which one spot was present only in follicular fluid whereas eight were found only in serum. The reason why some proteins are absent from follicular fluid could be due to either their degradation in follicular fluid or their inability to pass through the blood-follicle barrier and to enter into the antrum. In addition, 21 spots were down-regulated in follicular fluid in comparison to serum (e.g. alpha-2-macroglobulin, ceruloplasmin and antithrombin-III). All of these proteins are produced by the liver and belong to the acute phase proteins family. Nevertheless, de novo expression of α2-macroglubulin by granulosa cells has been previously demonstrated [23,24]. Furthermore, antithrombin-III is necessary to block the coagulation process in follicular fluid and to maintain the fluidity of follicular fluid until the release of the oocyte at ovulation [25].

### Immunodepletion/enrichment of mare follicular fluid prior to proteomic analysis

Almost all of identified proteins belonged to high-abundance serum proteins. We hypothesized that the depletion of such proteins may improve the resolution and the detection of low-abundance proteins. To our knowledge, no method had yet been developed to deplete the follicular fluid protein composition prior to its proteomic analysis. For this purpose, we compared the efficiency of four Immunodepletion columns known to be effective in filtering out some high-abundance proteins from human serum. These columns were based on either IgY (ProteomeLab IgY-HSA and ProteomeLab IgY-12) or on IgG (MARS-6 and ProteoPrep 20) [26-29]. Despite the similarity between these two types of antibodies, there exist some differences in their chemical structures [30]. According to our results of protein assays and 2D-PAGE, IgY columns demonstrated a higher depletion efficiency than IgG columns. This could be explained by the fact that IgY antibodies are less species-specific than IgG, and thus display a higher binding capacity to bind mare proteins. Moreover, we showed that ProteomeLab IgY-HAS and Mars-6 were more efficacious in removing albumin and immunoglobulins from mare follicular fluid than the two other columns. Nevertheless, the decrease of albumin in depleted fractions failed to reveal any new protein spot on 2D-PAGE patterns. This result was in accordance with the study by Echan et al. (2005) showing that 2D-PAGE analysis of depleted human plasma by MARS-6 failed to reveal any low-abundance protein [31].

In order to identify some proteins after depletion by MARS-6 column, 18 protein spots were excised from 2D-PAGE. From that number, 14 were identified positively which correspond to 12 unique proteins. All of these proteins except one were initially identified in crude mare follicular fluid and belong to high-abundance proteins. Of note that histidine-rich glycoprotein was identified from an excised spot in the region initially occupied by albumin, which clearly confirmed the potential masking effect of albumin as a high-abundant protein.

Although the Immunodepletion strategy succeeded in removing selectively high-abundance serum proteins from various biological fluids [29,32], it failed to deplete the mare follicular fluid under our experimental conditions, since it did not increase the list of identified proteins. One major limitation of this approach is the dilution of proteins into high salt buffers, which may reach a 100 fold ratio compared to the original sample. Thus, additional handling steps of concentration are required. In our study, we were successful in using the recommended ultra-filtration technique to concentrate samples (protein recovery of about 75-85%, data not shown). Nevertheless, this was time consuming procedure which may lead to the loss of some proteins. In our opinion, another limitation of the Immunodepletion strategy is related to potential interaction between some of high-abundance proteins and other factors, since the Immunodepletion steps have to be carried out under non-denaturing conditions. Thus, the depletion of proteins like albumin may lead to the loss of many other compounds including some low-abundance proteins [33-36]. An additional step of protein-protein interactions dissociation during sample preparation should overcome this limitation.

In parallel with the Immunodepletion strategy, we also evaluated the efficiency of hexapeptide ligand library columns, always with the aim to increase the detection of low-abundance proteins by 2D-PAGE. This method is based on affinity chromatography, where the complex biological sample containing high and low-abundance proteins (here the follicular fluid) is exposed to a very large number of hexapeptide ligands [37]. Hexapeptide ligand library column enrich low-abundance proteins while concomitantly reducing the concentration of high-abundance proteins [38], according to the principle of saturation-overloading chromatography. In addition, some high-abundance proteins like albumin and immunoglobulins are equalized, and not totally removed after enrichment.

In order to test the hypothesis that the hexapeptide ligand strategy may improve the detection of low-abundance proteins in mare follicular fluid, we excised 37 proteins from 2D-PAGE either by localization or by intensity. This led to identification of 33 spots corresponding to 18 proteins in enriched follicular fluid. In fact, three of these proteins (ALB, APOA1, and FGA) had already been identified in crude follicular fluid. Hexapeptide ligand library column allowed us to enlarge the list of the identified proteins from 18 in crude follicular fluid to 31 after enrichment. New identified proteins were either cytosolic (ACT, SNX1, GAPDH, GPX) or secretory (AMBP, FCN1, CLU, C3, FGG, FGB, FH and PSP). Of note is the fact that secretory proteins belong to the acute phase proteins family, and normally originate from serum. The role of cytosolic proteins like GAPDH and ACT is not clearly demonstrated in reproduction. We hypothesize that the presence of actin in follicular fluid could be related to changes in microfilaments and degradation of extracellular matrix during the preovulatory phase.

Ficolin-1 was demonstrated in mare follicular fluid, whereas ficolin-3 was previously identified in human follicular fluid [9]. This protein activates the complement factor and plays a role in the immune system. Furthermore, three proteins (Fibrinogen β, complement regulator factor H and AMBP) were identified in the region initially occupied by heavy and light chains of immunoglobulins. Altogether, our study showed that the combination of hexapeptide ligand library column and 2D-PAGE led to an improvement in the resolution of 2D-PAGE since gels exhibit many more protein spots, in the entire pH interval, than with crude samples. In addition, new proteins were identified in follicular fluid after enrichment.

The shotgun approach used in this study revealed that 24 proteins were identified only in reduced conditions whereas 25 were identified only in unreduced conditions. The use of this approach in combination with hexapeptide ligand library column allowed us to increase the list from 31 to 113 unique proteins in mare follicular fluid. Again, some of them were high-abundance proteins whereas several may play a significant role during folliculogenesis. For example, afamin is a glycoprotein with vitamin E-binding properties may be involved in ovarian activity and function since vitamin E plays an important role in ovarian steroidogenesis [39]. More recently, Jackson et al. (2007) showed a significant decrease in total afamin concentration in serum of patients with ovarian cancer, compared to healthy controls [40]. This protein has already been identified in human follicular fluid [8]. Annexin-I (ANX1) was identified in this study whereas only annexin IV (ANX4) was previously reported in the human follicular fluid [8]. In addition, five various isoforms of annexin (ANX1, ANX 2, ANX4, ANX 5 and ANX 11) have already been identified in human ovary [41]. This protein is part of a group calcium/phospholipid-binding proteins involved in regulation, proliferation, exocytosis and membrane fusion [42]. Another group of proteins that we identified in the follicular fluid correspond to extracellular matrix proteins, which are known to play important roles in cell-matrix interactions, matrix assembly and wound healing [43]. Dermatopontin is a widely distributed small molecular weight protein, assumed to be involved in wound healing. One can hypothesize that the two latter proteins are functionally involved in the repair of the follicular wall after ovulation. Vitronectin has previously been demonstrated in bovine follicular fluid and its concentration varies with the stage of follicle development [18]. Although the role of this protein in ovarian follicular development is not known, it may play a role in follicular growth, selection of the dominant follicle and the ovulation process, due to interactions with other related glycoproteins (fibronectin, laminin, etc.).

In the present study, extracellular matrix protein 1 (ECM1) was also identified in the follicular fluid. ECM1 is a secretary glycoprotein suggested to play a role in angiogenesis [44]. The anticoagulant heparan sulfate proteoglycan has previously been found in rodent granulosa cells and in human follicular fluid. It probably plays a role in the rupture of the ovarian follicle at the time of ovulation [45]. Finally, glutathione peroxidase has already been shown in human follicular fluid [46,47] and has been suggested to protect the organism from oxidative damage.

### Annotation of all identified proteins in mare follicular fluid

Finally, we carried out subcellular localization and molecular function classification of all the proteins identified in this study (n = 113). We demonstrated that the majority (83%) was localized at the extracellular region, which implies that these proteins either come from the circulation system or are secreted by the follicle cells. Surprisingly, the rest were localized in the intracellular position like cytoplasm, cytoskeleton, membrane or nucleus. This finding is entirely in keeping with the hypothesis that some of follicle cells are being damaged during follicle development, leading to the release of their cellular component into the follicular fluid. Some evidences suggest that the formation of follicle cavity may result from the death of granulosa cells. If so, follicular fluid may fill the space left with DNA providing the osmotic force [48]. In addition, the procedure of follicular fluid collection probably could lead to some cellular damage.

The functional annotation showed that the proteins identified in this study were involved in 18 groups of molecular functions. Most proteins were classified in immunity and defense (14%), complement system (9%) or blood coagulation (8%) categories. All of these proteins are crucial components in inflammatory responses. Twelve proteins belong to the family of the complement factor with their inhibitors (factor I, Factor H). Recently, Jarkovska et al. (2010) showed the involvement of innate immune function of complement cascade proteins in human follicular fluid, and suggested a possible link to angiogenesis which is a vital process in folliculogenesis [9].

It is known that blood coagulation proteins are involved in both intrinsic (contact activation pathway) and extrinsic (tissue factor) pathways. Yamada et al. (1995) described only the intrinsic pathway proteins involvement in mare follicular fluid [49]. These proteins lead to fibrin formation and coagulation of follicular fluid. In the present study, we also identified inhibitors of coagulation, like antithrombin-III, which helps the follicular fluid to keep its fluidity during the transfer of the oocyte from the follicle to the oviduct.

Proteins involved in development and function of the reproductive system represent only 2% of our identified proteins. These were various fragments of inhibins (alpha and beta) and angiotensinogen. Inhibins are produced by granulosa cells in response to FSH stimulation that play a role in inhibiting FSH synthesis and secretion [50]. It has already been shown in follicular fluid of various species by using immune-detection or biological assays [51,52] but its identification by a proteomic approach is unique, and shows the power of the methods we used in this study.

Angiotensinogen was classified as a protein involved in the development and function of the reproductive system. Besides the well-described role of angiotensinogen in the renin-angiotensin system (RAS) regulating vasopressor, electrolyte, and fluid homeostasis [53], increasing evidence suggested a role for angiotensinogen in embryonic development. This protein has already been identified in follicular fluid of human [8] and porcine [54], as well as in rat granulosa cells [55].

### Description of an overlapping mare follicular fluid protein set shared with three other mammalian species

One may regard the follicular fluid proteome as made up of two parts: (1) a fixed set of proteins ("core proteome"), which composition does not vary, and which should be present in follicular fluid irrespective of species or/and the analytical methods used, and (2) a variable set of proteins, which abundance is dependent on several physiological and experimental factors. Taking this consideration, we compared our results with those from three previous comprehensive proteomic studies of the follicular fluid which have been performed in three different species including canine, human and porcine [8,9,11,12]. Using this comparison, the list of identified proteins in the follicular fluid is increased from 113 to 154. Twelve of them (ALB, AHSG, SERPINC1, APOA1, CLU, C4A, CFB, FGG, HP, RBP4, TF and GC) were identified in all studies, regardless of the species studied, whereas 17 other were present in at least three out of the four species studied. The list of species specific proteins (MFF = 60, HFF = 20, PFF = 13 and CFF = 5) showed that our proteomic method is more powerful to identify follicular fluid specific proteins. The closer look at these species specific proteins showed that some of them were different fragments of high-abundance proteins like immunoglobulins, complement factors and apolipoprotein, and this diversity could be due to various experimental procedures on the sample. In addition, the proteins related to reproduction (inhibins) were identified only after enrichment of mare follicular fluid. Furthermore, we found several new proteins in mare follicular fluid but their possible role in reproduction has not been investigated.

## Conclusions

This study provides the first description of mare follicular fluid proteome during the late follicle development stages. Our result of protein identification showed that the majority of proteins are high-abundance proteins, previously identified in the serum. Although the comparison of the proteins profiles of follicular fluids collected at three physiological stages revealed no differential proteins, the comparison between follicular fluid and serum revealed 30 differentially expressed proteins. In addition, we showed that enrichment method was a powerful tool compared to Immunodepletion of follicular fluid before its proteomic analysis. Based on our results, we conclude that the enrichment method can be used in combination with 2D-PAGE and mass spectrometry to visualize and further identify the low-abundance proteins in follicular fluid.

## Methods

### Animals, monitoring of oestrous cycle and treatment

Cyclic Welsh pony mares (3-19 years old, n = 12) from our experimental herd (INRA, Nouzilly, France) were used in this study. They were in good body condition, kept indoors, fed with concentrates (1.8 kg/mare/day) and had free access to water and trace-mineralized salt. Ovarian activity was assessed by routine daily transrectal ultrasonic imaging. Mares were randomly divided into three groups (see collection of mare follicular fluid). Mares from the third group received an i.v. injection of crude equine gonadotropins (CEG; 15 mg i.v.) when the largest follicle reached 33 mm in order to induce preovulatory maturation [56]. All procedures used for mare follicular fluid collection (this study) were approved by the agricultural and veterinary research agencies (approval number C37-175-2/37-035) and conducted in accordance with the guidelines for Care and Use of Agricultural Animals in Agricultural Research and Teaching.

### Collection of mare follicular fluid

Follicular fluid samples were collected at three different follicle development stages. In the first group of mares, punctures were performed at the early dominance stage (n = 3), when the largest follicle was between 22 and 25 mm in diameter. In the second group of mares, punctures were performed at the late dominant stage(n = 3), when the largest follicle reached 33 mm in diameter. The third group of mares was punctured at the preovulatory stage (n = 3), 34 hours after CEG injection. Follicular fluid samples were aspirated by transvaginal ultrasound-guided follicular puncture with a 7.5 MHz electronic convex transducer (Aloka SSD-900) equipped with a sterile single lumen needle (60 cm-long, 1.8 mm outer diameter), as previously described [56,57]. During each puncture session, blood samples were also collected from the jugular vein for serum preparation. Follicular fluids and serum were centrifuged for 10 min at 3000 g and individually frozen at -80°C until further processing. Only follicular fluids free from blood contamination were kept for further analysis.

### Immunodepletion and enrichment of follicular fluids

Four different human serum Immunodepletion columns were used to remove high-abundance proteins from mare follicular fluid. The names and properties of these columns are presented in Additional file 1. In addition, hexapeptide ligand library column (One-Step Elution) was also used to enrich crude mare follicular fluid [58]. Briefly, hexapeptide ligand library columns were washed two times (each 5 min) with water, then with wash buffer. Thereafter, 1 ml of crude FF was applied to each column and incubated for 2 h. The columns were then washed 3 times (each 5 min) with wash buffer. Finally, proteins bound to hexapeptides were eluted by 3 × 100 μL of elution reagent (enriched FF). All depletions and enrichment steps were performed at room temperature as recommended by the manufacturer.

### Protein assay and gel electrophoresis of mare follicular fluid and serum

All chemicals and materials used for proteomic analysis were purchased from Bio-Rad (Marnes-la-Coquette, France), unless otherwise indicated.

#### Protein assay

The total protein content of crude, depleted and enriched mare follicular fluid samples was determined using Bio-Rad DC (detergent compatible) assay with bovine serum albumin as standard (Pierce, Rockford, IL), and statistically analyzed by the non-parametric Kruskal-Wallis test. Before electrophoresis, the depleted and enriched fractions were concentrated by 10 kDa molecular mass cut-off centrifugal concentrators (Microcon^®^, Millipore, Bedford, MA, USA) and the pH of fractions was neutralized by 50 mM Tris PH 8.8.

#### 1D-PAGE

Enriched fractions (15 μl) of mare follicular fluid were diluted with an equal volume of buffer (Tris-HCl 160 mM pH6.8, EDTA 10 mM, SDS 10%, Glycerol 20%, bromophenol blue) in reduced (with β-mercaptoethanol 10%) and unreduced (without β-mercaptoethanol 10%) conditions. The samples were boiled for 4 min before being loaded into each lane. Proteins were separated using the NuPage system (Invitrogen, Cergy Pontoise, France) with 4-12% Bis-Tris gels. Molecular weight standards were also routinely loaded. Electrophoresis was performed at a constant intensity of 200 V. At the end of migration, gels were stained with 0.2% Coomassie Brilliant blue (CBB) (R350). In both conditions (reduced/unreduced), lanes were cut in 21 bands and processed for protein identification by mass spectrometry.

#### 2D-PAGE

The 2D-PAGE analysis was performed as previously published by Fahiminiya et al. (2010) [11,58]. Briefly, 100 μg protein samples were diluted with hydration solution containing urea (8 M), thiouree (2 M), CHAPS (4% w/v), ampholytes (0.2%) and dithiothreitol (DTT, 20 Mm), for isoelectric focussing (IEF). This solution was actively absorbed (50 V, 20°C, 11-16 h) into ReadyStrip™ IPG (11 cm IPG strips, pH range 3-10). IEF was performed in Protean Isoelectric Focusing System using the following conditions: 400 V for 4 h, 4000 V for 13 h, and 500 V for 14 h. Before carrying out second dimensional SDS-PAGE, the strips were equilibrated twice, each time for 15 min, with equilibration buffer (50 mM Tris-HCl pH 6.8 containing urea (6 M), glycerol (20% v/v), SDS (2% w/v)). During two steps of equilibration, first DTT (2%), then, iodoacetamide (2.5% w/v) was added to equilibration buffer. Equilibrated IPG strips were subjected to SDS-PAGE (10%). Gels were placed on running buffer containing (0.25 M) Tris Base, (2 M), glycine and (1%) SDS and was conducted as follows: 10 mA/gel for 45 min and 200 V for 5-6 h. After electrophoresis step, 2D-PAGE gels were stained with a mass spectrometry (MS) compatible silver nitrate. Three 2D gels were performed for each group.

#### 2D-PAGE image analysis

Stained gels were scanned and digitalized at 16-bit resolution using ImageScanner (Amersham Pharmacia Biotech, GE Healthcare Europe GmBH, Orsay, France). The resulting TIFF images were analyzed with Progenesis software (version 2008; Nonlinear Dynamics Ltd, Newcastle upon Tyne, UK), as previously described [58]. Using Progenesis, the automatic analysis protocol for gel images included spot detection, warping, background subtraction, average gel creation, matching, and reference gel modification. Each spot volume was normalized by volume per area ratio. Differentially expressed protein spots were determined using analysis of variance (ANOVA) which was included in the Progenesis software. The protein spots with a p-value less than 0.05 were considered as significant and those spots were subsequently cut from 2D gels and identified through mass spectrometry (MS).

#### Identification of proteins by mass spectrometry

Spots and bands of interest were cut into small blocks. Gel blocks were rinsed with water and acetonitrile before being reduced with dithiothreitol (DTT) and alkylated with iodoacetamide. They were incubated overnight at 37°C in 25 mM NH4HCO3 with 12.5 ng/μl (CBB) or 6.25 ng/μl (silver) (Sequencing Grade, Roche, Paris) as described by Shevchenko et al. (1996) [59]. The tryptic fragments were extracted, dried, reconstituted with 0.1% formic acid, and sonicated for 10 min. Nanoscale capillary liquid chromatography-tandem mass spectrometry (LC-MS/MS) using Q-TOF mass spectrometers was used to sequence the tryptic fragments as previously published by Belleannee et al. (2011) [60].

The peptide and fragment masses obtained were matched automatically to proteins in non-redundant database (NCBI all taxa) using the MS/MS ion search option of the MASCOT software http://www.matrixscience.com. For the database search, 2 tryptic missed cleavages were allowed and carbamidomethylcysteine and methionin sulfoxide were set as a variable modifications. The mass tolerance was 0.3 Da for both precursors and fragment ions. Protein hits were validated if the protein scores were above the MASCOT default significance threshold (p < 0.05).

### Functional and sub-cellular classification of mare follicular fluid proteins

Functional classification of proteins was achieved using a multi-staged classification methodology based upon three different databases: 1) Gene Ontology http://www.geneontology.org 2) the "DAVID" database http://david.abcc.ncifcrf.gov and 3) UniProt http://www.uniprot.org. Proteins which remained unclassified after applying the three above mentioned tools were placed in the "not determined" (ND) category. Classification of proteins according to their cellular localization was achieved using UniProt and LOCATE Database http://locate.imb.uq.edu.au. Again, unclassified proteins were placed in the ND category.

## Competing interests

The authors declare that they have no competing interests.

## Authors' contributions

SF was involved in the design of the study and performed sample collections, depletion, enrichment and the 2D-PAGE experiments on mare follicular fluid. She also computerized image analysis, analyzed the mass spectrometry data, performed bioinformatic analysis and drafted the manuscript. VL identified proteins by mass spectrometry. SR was provided the depletion columns and helped for Immunodepletion of follicular fluid. JLD assist to design the study and helped for follicular fluid enrichment. NG design and supervised the study, performed sample collection and depletion of follicular fluids, and revise the manuscript. All authors read and approved the final manuscript.

## Supplementary Material

Additional file 1 **Supplemental Table 1: Major Characteristics of the evaluated Immunodepletion columns**. Click here for file

Additional file 2 **Supplemental Figure 1: Silver stained 2D-PAGE profile of depleted mare follicular fluid by four depletion columns:** 100 μg of proteins samples were applied to a non-linear IPG strip (pI 3-10) in the first dimension and separated on SDS-PAGE (10%) gel in the second dimension (Molecular weight: 10-200 kDa range). The positions of some high-abundant proteins like albumin (ALB), immunoglobulin heavy chain (IGHC1) and Apolipoprotein A-I (APOA1) are shown on the Figure. Click here for file

Additional file 3 **Supplemental Table 2: The list of identified mare follicular fluid protein under reduced condition after enrichment with hexapeptide ligand library column**. Click here for file

Additional file 4 **Supplemental Table 3: The list of identified mare follicular fluid protein under unreduced condition after enrichment with hexapeptide ligand library column**. Click here for file

Additional file 5 **Supplemental Table 4: Classification of all identified proteins in mare follicular fluid using diverse proteomic approaches**. Click here for file

Additional file 6 **Supplemental Table 5: Overview of all identified proteins in follicular fluid of four different species using diverse proteomic approaches**. Click here for file
