# Supplementary figures and images for: Proteomic analysis of mare follicular fluid during late follicle development
Source: Proteome Sci. 2011 Sep 17;9:54. doi: 10.1186/1477-5956-9-54 (PMC3189114; doi:10.1186/1477-5956-9-54)

## Slide 1
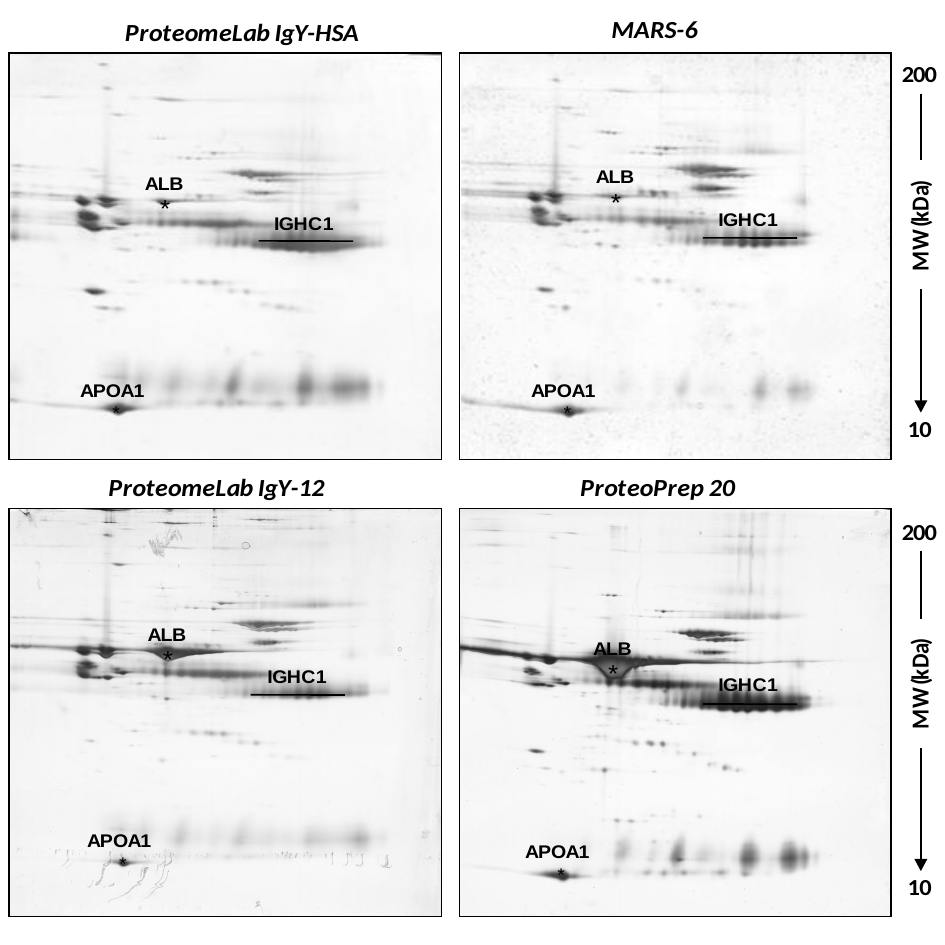

Supplement: Additional file 2 — Supplemental Figure 1: Silver stained 2D-PAGE profile of depleted mare follicular fluid by four depletion columns: 100 μg of proteins samples were applied to a non-linear IPG strip (pI 3-10) in the first dimension and separated on SDS-PAGE (10%) gel in the second dimension (Molecular weight: 10-200 kDa range). The positions of some high-abundant proteins like albumin (ALB), immunoglobulin heavy chain (IGHC1) and Apolipoprotein A-I (APOA1) are shown on the Figure. [file 1477-5956-9-54-S2.PPT]
